# Audiovisual Integration Enhances Stimulus Detection Performance in Mice

**DOI:** 10.3389/fnbeh.2018.00231

**Published:** 2018-10-04

**Authors:** Guido T. Meijer, Jean L. Pie, Thomas L. Dolman, Cyriel M. A. Pennartz, Carien S. Lansink

**Affiliations:** ^1^Swammerdam Institute for Life Sciences, Center for Neuroscience, Faculty of Science, University of Amsterdam, Amsterdam, Netherlands; ^2^Research Priority Program Brain and Cognition, University of Amsterdam, Amsterdam, Netherlands

**Keywords:** multisensory, sensory, cross-modal, cue-integration, auditory, perception, behavior

## Abstract

The detection of objects in the external world improves when humans and animals integrate object features of multiple sensory modalities. Behavioral and neuronal mechanisms underlying multisensory stimulus detection are poorly understood, mainly because they have not been investigated with suitable behavioral paradigms. Such behavioral paradigms should (i) elicit a robust multisensory gain, (ii) incorporate systematic calibration of stimulus amplitude to the sensory capacities of the individual subject, (iii) yield a high trial count, and (iv) be easily compatible with a large variety of neurophysiological recording techniques. We developed an audiovisual stimulus detection task for head-fixed mice which meets all of these critical behavioral constraints. Behavioral data obtained with this task indicated a robust increase in detection performance of multisensory stimuli compared with unisensory cues, which was maximal when both stimulus constituents were presented at threshold intensity. The multisensory behavioral effect was associated with a change in the perceptual performance which consisted of two components. First, the visual and auditory perceptual systems increased their sensitivity meaning that low intensity stimuli were more often detected. Second, enhanced acuity enabled the systems to better classify whether there was a stimulus or not. Fitting our data to signal detection models revealed that the multisensory gain was more likely to be achieved by integration of sensory signals rather than by stimulus redundancy or competition. This validated behavioral paradigm can be exploited to reliably investigate the neuronal correlates of multisensory stimulus detection at the level of single neurons, microcircuits, and larger perceptual systems.

## Introduction

The detection of stimuli is an important perceptual competence which animals must perform constantly to identify potential threats, food sources, and conspecifics. Arguably, stimulus detection is the most basic ingredient of perception, because it merely entails a behavioral judgment about the presence or absence of something regardless of its identity or properties (Pennartz, 2015). The accuracy and speed at which stimuli are detected have been shown to improve when multisensory signals arising from the same objects are combined (Gingras et al., 2009; Hirokawa et al., 2011; Gleiss and Kayser, 2012; Hammond-Kenny et al., 2017). These behavioral improvements are thought to be mediated by multisensory *cue-integration* on the neuronal level, which is a mechanism by which cues can provide a more reliable estimate of an external event as compared to cues from the same sensory modality (Fetsch et al., 2013). Multisensory cues, however, do not necessarily have to be integrated to achieve stimulus detection benefits (Lippert et al., 2007). In any situation in which two stimuli are presented instead of one, subjects can achieve a behavioral gain by independently judging either one of the two stimuli, without any interaction in the brain between the two involved sensory systems. Although this “stimulus redundancy effect” has been widely described in human behavioral studies (Miller, 1982; Forster et al., 2002; Gondan et al., 2005; Rouger et al., 2007), it is mostly ignored in animal studies (but see Gingras et al., 2009) and it is unclear to what extent this alternative mechanism can explain multisensory stimulus detection benefits.

Detection performance improves when a stimulus is composed of features from multiple sensory modalities, but at which relative intensity levels is this detection improvement the strongest? The “law of inverse effectiveness” describes that the magnitude of multisensory benefit gets progressively larger as the effectiveness by which stimuli drive the sensory systems decreases (Meredith and Stein, 1983, 1986; Serino et al., 2007; Gleiss and Kayser, 2012). This law predicts that the multisensory benefit is largest when the individual stimuli are faint or difficult to distinguish from the background. Systematically testing this prediction, however, has proven difficult because it requires a direct comparison between stimulus intensities of different sensory modalities and sampling of many combinations of stimulus intensities (i.e., necessitates a large number of behavioral trials performed by the subject). Thus, it remains unclear at which combination of stimulus intensities the highest behavioral gain is achieved.

A currently unexplored question is how multisensory integration can improve the ability to detect a stimulus. At least two possible contributing factors can be distinguished: sensory systems can improve their sensitivity or their acuity. The former results in a lowering of the detection threshold for sensory signals which are composed of features from multiple modalities, this enables the detection of faint stimuli. The latter is reflected by a steepening of the slope of the psychophysical function which results in a reduction of the range of stimulus intensities in which not every stimulus is detected. It is unclear which of these mechanisms, which are not mutually exclusive, contribute to multisensory behavioral gain.

Understanding the behavioral and neuronal mechanisms which govern signal detection performance requires adequate behavioral paradigms which probe stimulus detection by systematically calibrating the stimulus amplitude to the sensory capacities of the individual subject, and with a high trial count for reliable tracking of behavioral and neuronal read-outs. We present a novel audiovisual stimulus detection task for head-fixed mice which meets these critical behavioral constraints. With data derived from this behavioral paradigm we addressed the following research questions: (i) Is audiovisual stimulus detection dependent on cue integration? (ii) At which combination of stimulus amplitudes is the largest behavioral benefit achieved? (iii) Which factors in sensory processing underlie these behavioral benefits?

## Materials and Methods

### Mice

All animal experiments were performed according to the national and institutional regulations. Male C57Bl/6 mice were obtained from Harlan Sprague Dawley Inc., and housed socially (groups of 2–4) in cages provided with cage enrichment. To perform behavioral testing in the active phase, mice were kept at a 12 h reversed day-night cycle (lights on 8pm). The age of the mice (*n* = 7) ranged between 96 and 209 days at the first day of behavioral testing.

### Headbar Implantation Surgery

Mice were studied under head-fixed conditions providing a stable alignment between subject and hardware by which the spatiotemporal arrangement of stimulus presentation with respect to the relevant sensory organs can be controlled. To allow head fixation during behavioral training a titanium head bar (length: 28 mm; width: 10 mm) was implanted on the skull. After a subcutaneous injection of the analgesic buprenorphine (0.025 mg/kg) and under isoflurane anesthesia (induction at 3%, maintenance at 1.5–2%) the skin above the skull was epilated, disinfected, and an incision was made. The head bar was attached to the exposed skull using C&B Super-Bond (Sun Medical, Japan). Skin surrounding the implant was covered using tissue adhesive (3M Vetbond, MN, United States) preventing post-surgical infections. Mice were allowed to recover for a week after implantation.

### Training Apparatus

**FIGURE 1:**
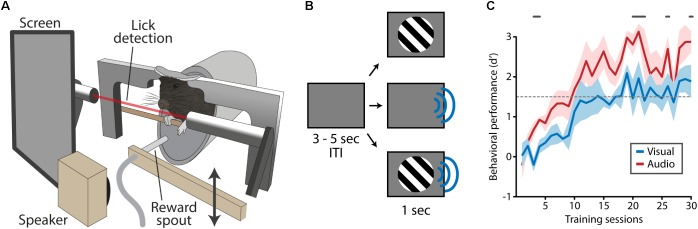
Training apparatus and task design. **(A)** Mice were positioned in the training apparatus with their heads fixed and their body in a body holder. Visual stimuli were presented on a screen located in front of the right eye, auditory stimulation was delivered through a speaker next to the screen. An infra-red beam in front of the mouth enabled the detection of licks. A reward spout, which was out of reach during most of the trial, was moved toward the mouse’s mouth at times reward was dispensed. **(B)** A trial started with an inter-trial interval (ITI) during which a gray isoluminant screen was shown. Following the ITI, visual, auditory or audiovisual stimuli were presented. **(C)** During task acquisition mice were trained to respond to visual and auditory stimuli (no audiovisual stimuli) which typically took 15–20 days after which task performance was on average above the performance criterion of *d′* = 1.5 (dashed gray line). Mice responded with more fidelity to auditory compared to visual stimuli as indicated by a significantly higher d-prime (gray lines above plot indicate significant differences, Matched Pairs Signed Rank Test, *p* < 0.05).

Mice were fixated in a custom-built holder to which the implanted head-bar was firmly attached with small screws (**Figure 1A**; Meijer et al., 2017). The body of the mouse was put in a small tube to limit body movements. A bar was positioned in front of the mouse on which it could rest its front paws. The holder was placed in a dark and sound-attenuated cabinet. An infra-red beam was positioned in front of the mouth of the mouse to detect licking responses. When a lick response was detected, a spout was elevated by a servo and 6–10 μl of liquid reward (baby milk) was pushed through the spout by a motorized pump. After 2 s the reward spout was lowered to the base position so that it would remain out of reach of the mouse. Visual stimuli were presented by a 15” thin-film-transistor monitor with a refresh rate of 60 Hz, which was positioned 16 cm from the right eye and oriented at a 45-degree angle from the midline of the mouse. Auditory stimuli were amplified (RX-V44, Yamaha, Japan) and presented from a tweeter (DX25TG09-04, Vifa, Denmark) located in front of the mouse (distance: 28 cm).

### Visual and Auditory Stimuli

Visual stimuli consisted of square-wave gratings, with a temporal frequency of 1 Hz and spatial frequency of 0.05 cpd. These were presented in three different orientations (90, 210, 330 degrees) in varying contrast (0.5–15% for staircase trials, 100% for full contrast). To prevent an edge effect, the surround of the stimulus was a cosine-tampered circular window with a diameter of 60 retinal degrees. Auditory stimuli consisted of frequency modulated tones with a carrier frequency of 15 kHz and a peak frequency deviation from the carrier frequency of 1 kHz. The visual and auditory stimulus constituents were always temporally congruent, meaning that the temporal frequency of the visual stimulus and the modulation frequency of the auditory stimuli were both 1 Hz. During the inter-stimulus interval an isoluminant gray screen was presented.

### Behavioral Training Paradigm

Mice were motivated to perform the behavioral task by a water rationing paradigm. They typically earned their daily ration of liquid by performing the behavioral task but received a supplement when the earned amount was below the absolute minimum of 0.025 ml/g body weight per 24 h. Training generally consisted of four stages. In the first training stage, mice learned stimulus-reward associations through classical conditioning. Trials started with the presentation of a visual- or audio-only stimulus for 5 s, after which a reward was delivered automatically. Stimuli of each type were presented in blocks of 20–40 consecutive trials, and trials were separated by an inter-trial interval randomly set between 3–5 s. When mice made a licking response during the stimulus, reward was dispensed immediately without interrupting stimulus presentation. In several days, mice learned to reliably trigger reward delivery by licking during the stimulus presentation (hit rate >80%) and were advanced to the next stage.

In the second stage, mice could only obtain reward when they responded during the stimulus presentation. Because making false alarms was not punished, mice would often lick continuously regardless of stimulus presentation. This strategy was discouraged by only starting a new trial when no licking was detected in a “no-lick” window which consisted of the last 1–3 s (chosen randomly each trial) of the inter-trial interval. Stimulus duration was shortened from 5 to 3 s and blank trials, in which no stimulus was presented, were introduced to test whether responses were selective to the stimulus. Blank trials can be alternatively labeled “catch trials.” The stimulus selectivity of licking responses was tracked by computing the sensitivity index *d′* which is the difference between the false alarm rate and the hit rate.

(1) $$d^{\prime }=\phi^{-1}(HR)-\phi^{-1}(FAR)$$

Where Φ^-1^ is the inverse of the cumulative normal distribution of the hit rate (HR; rate of responses during stimulus trials) and the false alarm rate (FAR; rate of responses during probe trials). When mice showed a performance of *d′* > 1.5 for both visual and auditory trials they were advanced to the next stage of the behavioral paradigm.

In the third training stage, stimulus duration was shortened to 1 s and visual and auditory stimuli were no longer presented in blocks but were presented interleaved according to a semi-random schedule such that no more than four stimuli of the same modality were presented sequentially. Mice were advanced to the final task when their performance was *d′* > 1.5 for both visual and auditory trials for three consecutive days. Mice typically took 3–4 weeks to reach this point.

In the final task, the amplitude of audio- and visual-only stimuli was continuously adjusted around the perceptual thresholds for the mice. In addition, compound audiovisual stimuli were interleaved with unisensory stimuli. Separate adaptive staircase procedures were used for visual and auditory stimuli (PsychStairCase of the Psychophysics Toolbox for MATLAB; Brainard, 1997). The staircase was an adaptive Bayesian method to acquire both the threshold and the slope of the psychometric function. The procedure constructed an internal estimate of the psychometric function during the ongoing session and for each trial presented the stimulus amplitude which would provide the most expected information gain regarding the threshold and slope values (Kontsevich and Tyler, 1999). Because the adaptive method was built to acquire both the threshold and the slope it would not converge on the threshold which would result in an oversampling of the threshold intensity, instead it presented stimulus intensities around the inferred detection threshold. In practice this resulted in relatively big steps when the staircase inferred that the slope was shallow and small steps when the inferred slope was steep. Audiovisual trials were semi-randomly interleaved with the visual and auditory staircase trials. During a compound stimulus trial, the intensities for the visual and auditory components were copied from the last presented visual and audio-only trials. In this manner, a wide range of combinations of stimulus intensities was tested. Furthermore, in each session maximum intensity visual, auditory, and audiovisual trials (100% visual contrast/70 dB auditory amplitude) were presented for estimating the lapse rate (i.e., the failure rate at full contrast/high volume stimuli) of the performance. In summary, a behavioral session consisted of a semi-random sequence of visual-only staircase trials (25%), audio-only staircase trials (25%), audiovisual staircase trials (25%), 100% contrast visual-only trials (6.25%), 70 dB audio-only trials (6.25%), high intensity audiovisual trials (6.25%) and probe trials (6.25%).

To exclude periods in which the mouse was compulsively responding, the false alarm rate was calculated as the percentage of blank trials in which the mouse responded in a sliding window of 100 trials. This calculation started with the first trial window (trial 1–100) and advanced over the session (trial 2–101, 3–102, etc.). Trial blocks in which the false alarm rate exceeded 50% were excluded from the dataset, this amounted to 17.1 ± 7.6% excluded trials per mouse.

### Unisensory Psychometric Functions and Perceptual Thresholds

For the fitting of psychometric functions for the visual and auditory domain, trials were binned according to their stimulus intensity in three equally populated bins and the response rate was calculated for each bin. We opted for equally populated bins because binning by stimulus intensity would result in unequal number of trials in each bin. The visual contrast was log_10_ transformed to improve the fit of the visual psychometric function. The false alarm rate and the inverse of the lapse rate were added as extra bins at 0.1% contrast and 100% contrast, and 23 and 70 dB, respectively. Equation 2 describes the psychometric function, which is a cumulative normal distribution with three free parameters, that was fitted for both the visual and the auditory domain:

(2) $$f(x)=\gamma +(1-\gamma -\lambda )\left(\frac{1}{2}\left[1+\mathrm{erf}\left(\frac{x-\mu }{\sigma \sqrt{2}}\right)\right]\right)$$

Here, the false alarm rate γ was fixed to the measured false alarm rate, whereas the lapse rate λ, mean μ, and standard deviation σ were free parameters. The perceptual threshold of the fitted psychometric function was determined as the stimulus amplitude corresponding with the midpoint between the lower and upper bound of the fitted response rate.

### Multisensory Psychophysical Performance

Differences in psychometric functions between uni- and bimodal stimulus conditions were quantified using logistic regression (Equation 3). A change in visual psychophysics was determined by comparing the psychometric function for visual-only trials with the psychometric function for all audiovisual trials of which the auditory component was below the auditory detection threshold. Conversely, the psychophysical performance in the auditory-only condition was compared with the auditory supported by visual condition.

(3) $$f(x)=\frac{1}{1+e^{-(\beta_{0}+x\;*\;\beta_{1})}}$$

The logistic function *f*(x) fitted the psychometric function by maximizing the probability that the constant ß_0_ and the intercept ß_1_ resulted in the observed dataset using the MATLAB *glmfit* function. The continuous independent variable was the visual or auditory amplitude per trial and the binary depended variable was a zero or a one depending on whether or not the animal made a response during that trial. The slope of the resulting fit was calculated as follows:

(4) $$m=\beta_{1}(f(x)(1-f(x)))$$

Here, the slope *m* is calculated from the fitted logistic function *f* and the intercept ß_1_. The x-axis coordinate of the perceptual threshold was used for calculation of the slope.

### Uni- Versus Multisensory Response Rate

For comparing the performance of the mice during uni- and multisensory stimulation, trials for each condition were binned into three bins: (i) trials in which the visual and/or auditory stimulus intensity was below its respective perceptual threshold (*Sub threshold*), (ii) trials where the stimulus intensity was around the perceptual threshold (bin boundaries: 15% below – 15% above threshold; *Around threshold*), and (iii) trials in which visual and/or auditory stimulus intensity was above the perceptual thresholds (*Supra threshold*). This approach was chosen so that the multi- versus unisensory detection rate can be compared for groups of stimuli with equal subjective intensity. Notably, these bins were different from the binning used in fitting the perceptual function because in this case a metric derived from the perceptual function was needed to perform the binning.

### Subjective Intensity Response Matrix

We designed a subjective intensity measure for assessing the response differences across stimulus combinations. This measure, which is a reflection of the probability that a stimulus will be detected given its amplitude, is defined as the response rate associated with a certain stimulus amplitude according to the fitted psychometric function. That is, a subjective intensity of 0.5 equates to the visual contrast or auditory amplitude at which the mouse detected 50% of the stimuli. The subjective intensity is computed separately for visual and auditory components of bimodal stimuli.

For each mouse, audiovisual trials were binned in an equally spaced 4 × 4 grid according to the combination of their subjective intensities for the audio and visual components, each running between the false alarm rate (0.21 ± 0.03) and 1. Subsequently, the mean hit rate across all trials in each of the 16 bins of the grid was calculated. The mean subjective intensity matrix across mice was obtained by averaging all response matrices of individual mice using a weighted average according to the number of trials in every bin per mouse. Multisensory increases or decreases in performance per subjective intensity bin were determined by subtracting the expected unisensory hit rate (defined as the maximal unisensory subjective intensity for that bin) from the observed audiovisual hit rate.

### Behavioral Models of (Multi)sensory Processing

The behavioral strategy that mice used in this stimulus detection paradigm was estimated using signal detection theory, as extensively described in (Jones, 2016). We fitted three models on our behavioral data, which predicted perceptual sensitivity for the multimodal condition based on the unimodal sensitivities (visual- and auditory-only *d′*) by using a multimodal decision variable defined as a function of the unimodal decision variables.

The *Or* model, which is described by Equation 5, states that the mouse observes the visual and auditory component of the multisensory stimulus separately and makes a response when *either* of the two stimulus components exceeds its threshold. Sensory noise is added to the visual and auditory components at an early stage and the thresholds (λ) are computed from the false alarm ratio. This model assumes that no audiovisual integration takes place, the decision variable for multisensory trials (DV_OR_) is simply either the visual decision variable (DV_V_) or the auditory decision variable (DV_A_) depending on which one of the two components has the highest intensity in any given multisensory trial. Even though no integration is assumed in this model it still predicts an increased sensitivity in the multisensory compared to the unisensory condition because, by law of probability, the likelihood of detecting any of two stimulus constituents is larger than detecting a single cue only.

(5) $$DV_{OR}=\max(DV_{V},\;DV_{A})$$

In the *Linear Sum* model (Equation 6) the multisensory decision variable (DV_L-SUM_) is a linear weighted sum (visual weight: *w_V_*, auditory weight *w_A_*) of the visual and auditory unisensory decision variables. Independent internal noise is added to the two inputs prior to the summation which reflects sensory noise in the visual and auditory systems.

(6) $$DV_{L-SUM}=DV_{V}*w_{V}+DV_{A}*w_{A}$$

The *Non-linear Sum* model is similar to the linear sum model but adds a multiplicative component to the summation (Equation 7). Here the multiplication factor γ determines the non-linearity of the summation. When γ = 0, the model equals the linear sum model; when γ < 0, the summation becomes sub-linear and when γ > 0 the summation of visual and auditory input is supralinear.

(7) $$DV_{NL-SUM}=DV_{V}*w_{V}+DV_{A}*w_{A}+\gamma *DV_{V}*DV_{A}$$

For each mouse, the models were compared using a cross-validation scheme. The behavioral data was randomly split into two sets of equal size, the first set was used to determine each models’ parameters and the second one to test the model’s goodness of fit. Each dataset was binned according to unimodal stimulus intensity into two bins: sub-threshold and supra-threshold intensity. For each multimodal combination of stimulus intensities (four bins in total), multimodal sensitivity was computed according to the three multimodal integration functions described above (Equations 5, 6, and 7). Decision variable distributions were assumed to be normally distributed. Predicted sensitivities were compared to the observed ones. The models’ goodness of fit was computed using variance accounted for (VAF; Morgan et al., 2008):

(8) $$VAF=1-\frac{RSS}{SST}$$

Where *RSS* is the residuals squared sum and *SST* is the sum of squared distances to the mean (Morgan et al., 2008).

### Statistics

Whether the data was normally distributed was tested using the Jarque–Bera test for normality (H_0_: the data are normally distributed). When this test was not significant (*p* > 0.05), a parametric test was used (*t*-test, ANOVA). Alternatively, when the test was significant (*p* < 0.05) we used a non-parametric equivalent. Throughout the manuscript averages are reported as mean ± standard error of the mean.

## Results

The goal of this study was to reveal the behavioral mechanisms of audiovisual cue detection in a mouse head-fixed paradigm which allows high throughput training and can be easily combined with large scale neurophysiological recordings (i.e., electrophysiology, optical imaging) and intervention of neuronal activity (i.e., optogenetics, chemogenetics; **Figure 1A**). Mice were trained to make a lick response when they detected a visual, auditory or audiovisual stimulus which was calibrated in intensity to the perceptual capacities of the individual mouse.

### Task Acquisition and Unisensory Perceptual Performance

In the acquisition phase of the behavioral paradigm, mice were presented with clearly perceivable visual or auditory stimuli (100% contrast/70 dB; **Figure 1B**). Mice (*n* = 7) learned to selectively respond to these stimuli in around 15 training sessions after which their performance reached plateau levels (**Figure 1C**). Their behavioral performance was significantly better for auditory trials compared to visual trials (gray line in **Figure 1C**; Matched Pairs Sign Rank test, *p* < 0.05).

**FIGURE 2:**
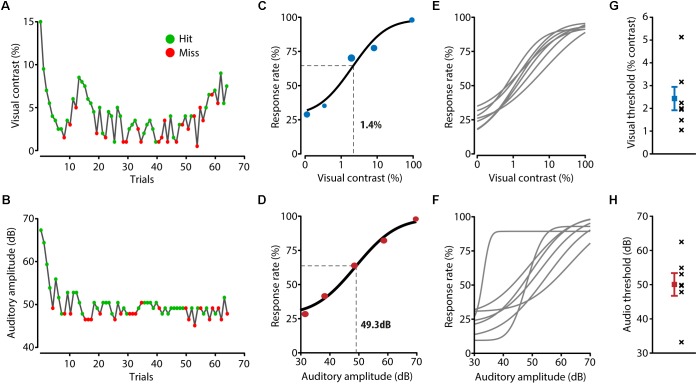
Detection performance for unisensory stimuli. **(A,B)** During a behavioral session, two adaptive staircase procedures were running in parallel for presenting visual **(A)** and auditory **(B)** stimuli around the perceptual threshold of the mouse. Green dots indicate trials in which the mouse licked in response to the stimulus, red dots indicate trials during which no response registered. **(C)** The visual perceptual threshold was determined by fitting a psychometric function to the response rate calculated for the binned visual stimulus contrast. Visual contrast was log_10_ transformed to allow for a better fit. The example mouse shown here has a visual threshold of 1.4%, determined as the point half way the fitted curve. **(D)** Example psychometric function and threshold calculation for auditory trials. **(E,F)** Fitted psychometric functions for all mice (*n* = 7). **(G)** Visual thresholds for all individual mice (black crosses) and the mean and SEM (in blue) over mice. **(H)** Auditory thresholds for all mice (black crosses) and the mean and SEM (in red) over mice.

In the final task, the amplitudes of the presented stimuli were calibrated around the perceptual threshold of the mouse using an adaptive staircase procedure (**Figures 2A,B**; see section “Materials and Methods”). This task included cross-modal stimuli, which were composed of the most recently presented stimulus amplitudes in the unisensory staircases, resulting in various combinations of visual and auditory amplitudes. Mice reliably performed many trials per day (325 ± 14) across multiple consecutive days (8 ± 1), resulting in a large number of trials in total (2004 ± 142) per mouse.

The perceptual thresholds for the visual and auditory modalities were determined as the stimulus amplitudes at the mid-point between the full range of the response rates of the fitted psychometric function (**Figures 2C–F**). Averaged over mice, the visual perceptual threshold was a contrast of 2.4 ± 0.5% and the auditory perceptual threshold was 50.1 ± 3.3 dB (**Figures 2G,H**) which is consistent with previous literature (Henry and Chole, 1980; Histed et al., 2012). Using logistic regression resulted in similar perceptual thresholds (visual: 1.2 ± 0.07%, auditory: 46.1 ± 1.9 dB).

### Multisensory Gain in Stimulus Detection Performance

**FIGURE 3:**
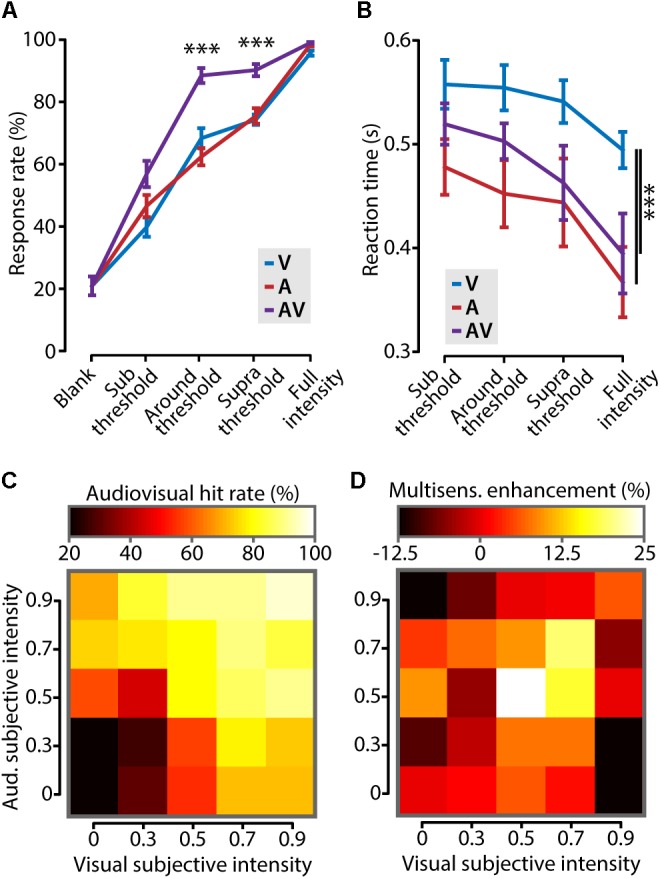
Cross-modal increase of stimulus detection performance. **(A)** Mice showed a significantly higher response rate (i.e., number of correctly detected stimuli as percentage of the total) in the audiovisual (AV) condition compared to the visual (V) and auditory (A) conditions when stimulus intensities were around and above (supra) threshold (one-way ANOVA with *post hoc* Tukey–Kramer; ^∗∗∗^*p* < 0.001). **(B)** Reaction times were shorter for stimuli that contained a tone (A and AV) compared to the visual-only condition (V, two-way ANOVA with *post hoc* Tukey–Kramer; ^∗∗∗^*p* < 0.001). **(C)** The performance on audiovisual trials for all different combinations of binned visual and auditory subjective intensity is color coded in a heat map. Subjective intensity is defined as the hit rate that a certain stimulus intensity elicits. The leftmost column and bottom row show the unisensory hit rates. **(D)** The increase in behavioral performance for all combinations of subjective intensities shows that cross-modal enhancement of behavior is strongest when both unisensory stimuli are presented at threshold intensity (subjective intensity ≈0.5).

We set out to investigate whether cross-modal stimuli elicited a behavioral gain compared to unimodal stimuli in this paradigm. For all stimulus types, mice showed progressively more accurate response behavior (**Figure 3A**; two-way ANOVA, *F*_2,90_ = 25.3, *p* < 10^-8^, *n* = 7) as well as shorter reaction times (**Figure 3B**; two-way ANOVA, *F*_2,54_ = 8.5, *p* = 0.0006, *n* = 7) with increasing stimulus amplitude. Mice responded with significantly higher accuracy to multisensory compared to unisensory stimuli when their intensities were around the perceptual threshold (difference in response rate: 25.2 ± 4.3%, one-way ANOVA, *F*_2,18_ = 33.9, *p* < 10^-5^, *n* = 7; **Figure 3A**). A similar behavioral improvement was found for stimuli of which the intensity was above threshold (supra threshold increase: 15.7 ± 2.7%; one-way ANOVA, *F*_2,18_ = 82.3, *p* < 10^-4^, *n* = 7), but not for subthreshold or full intensity stimuli. Besides improving detection accuracy, combining information from multiple sources has been shown to result in shorter reaction times (Gielen et al., 1983; Hirokawa et al., 2011; Gleiss and Kayser, 2012; Hammond-Kenny et al., 2017). In our task, mice responded significantly faster to auditory-only and audiovisual stimuli compared to visual-only stimuli (two-way ANOVA with *post hoc* Tukey–Kramer, *F*_2,54_ = 9.1, *p* = 0.0004, *n* = 7). The response times for visual stimuli are comparable to other studies testing stimuli of ∼1% contrast (Burgess et al., 2017). The reaction times for auditory and audiovisual trials were not significantly different, indicating that, in this behavioral paradigm, mice do not show a cross-modal facilitation of reaction times.

We next asked which combination of stimulus intensities elicited the largest cross-modal enhancement of behavioral performance. Stimulus intensities for auditory and visual stimuli, however, are not directly comparable because they are expressed in different dimensions (dB and % contrast, respectively). We solved this problem by transforming stimulus amplitudes for both modalities into “subjective intensities” which have no dimension. Subjective intensity is the response rate that corresponds to a certain stimulus amplitude. **Figure 3C** shows the hit rate for all combinations of auditory and visual subjective intensities. This matrix shows that the rate of correct responding progressively increases with stronger subjective intensities for both stimuli. Subsequently, the cross-modal enhancement, defined as the difference between the multisensory response rate and the maximal unisensory response rate, was calculated and color coded in the matrix shown in **Figure 3D**. The largest behavioral gain was achieved when the two stimulus components were of equal subjective intensity, and close to the perceptual threshold [White (0.5, 0.5) bin in **Figure 3D**]. In conclusion, cross-modal facilitation of stimulus detection is maximal when the two unisensory constituents are both presented around their respective perceptual thresholds.

### Multisensory Stimulation Improves Perceptual Sensitivity

We next asked which factors contribute to the multisensory gain in task performance. We considered two aspects of perceptual sensitivity of stimulus detection: sensitivity and acuity. The former is represented by the detection threshold such that a lower threshold shows an increased capability to detect stimuli. The latter is reflected in the slope of the psychometric function, a steeper slope is associated with an increased ability to classify whether a stimulus occurred or not. We investigated the facilitating role of a cross-modal stimulus on unisensory perceptual performance by comparing the psychometric functions, determined by logistic regression, from the unisensory conditions to the multisensory condition in which the cross-modal stimulus was below its perceptual threshold.

**FIGURE 4:**
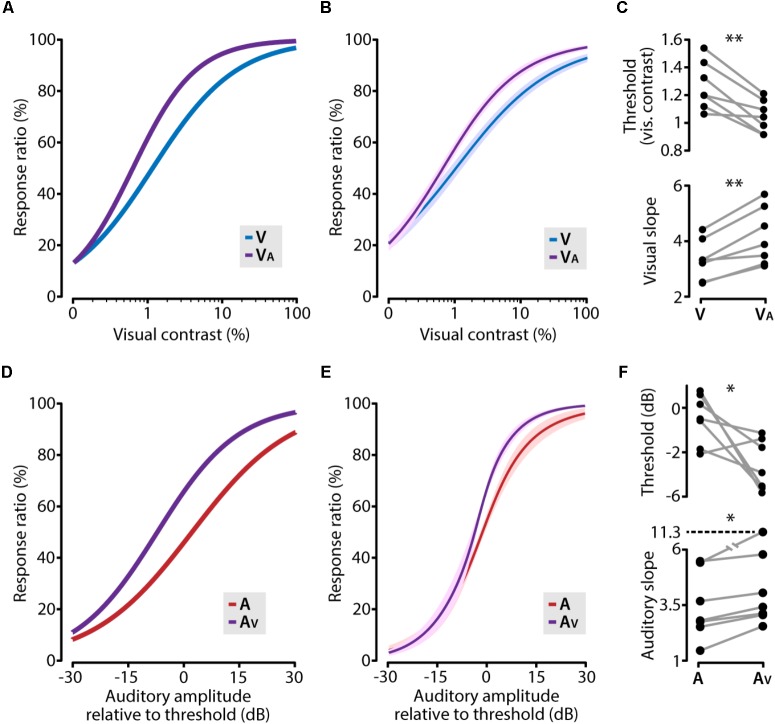
Cross-modal enhancement of perceptual sensitivity. **(A)** The visual psychometric function (V; blue line) of an example animal shifts toward lower contrasts when the visual stimulus was combined with a subthreshold auditory stimulus (V_A_; purple line). **(B)** The average psychometric function across all animals for visual (V; blue line) and visual supported by subthreshold auditory (V_A_; purple line). Solid line indicates the mean, shading SEM. **(C)** The facilitating role of a subthreshold auditory stimulus resulted in a significant decrease of the visual contrast threshold and a significant steepening of the psychometric function (paired *t*-test; ^∗∗^*p* < 0.01). **(D–F)** same as **(A–C)** but for auditory and auditory supported by sub threshold visual stimuli (paired *t*-test; ^∗^*p* < 0.05).

Concurrently presenting a subthreshold auditory stimulus together with a visual stimulus shifted the psychometric function toward lower visual contrasts compared to the unisensory visual function (**Figures 4A,B**). This was reflected in a significant reduction of the visual contrast threshold (paired *t*-test, *p* = 0.0028, *n* = 7) and a steepening of the slope (paired *t*-test, *p* = 0.0019, *n* = 7; **Figure 4C**). In the same vein, an auditory cue supported by a subthreshold visual stimulus resulted in a similarly shifted psychometric function (**Figures 4D,E**), including a significantly lower perceptual threshold (paired *t*-test, *p* = 0.046, *n* = 7) and a steeper psychometric function (Wilcoxon matched-pairs signed-ranks test, *p* = 0.016, *n* = 7; **Figure 4F**). In these analyses, we used auditory stimulus intensities relative to the auditory detection threshold, but using the absolute stimulus intensities yielded qualitatively similar results. These results indicate that the increased perceptual performance in the cross-modal condition consisted of two factors: mice showed a lower perceptual threshold and second, the sensitivity for small changes in contrast along the dynamic range was increased in the audiovisual condition as compared to the unisensory condition.

### Mice Integrate Visual and Auditory Input During Multisensory Stimulus Detection

**FIGURE 5:**
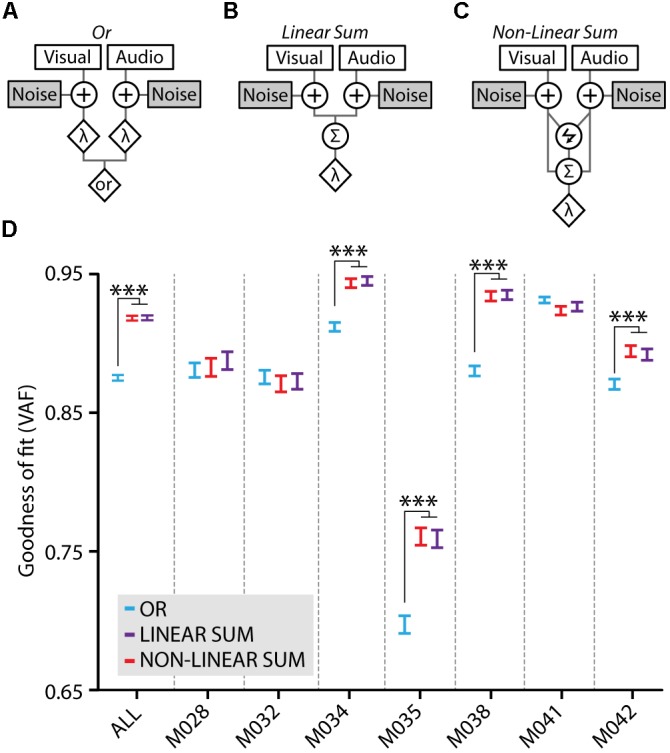
Model fits to the behavioral data. **(A)** The *Or* model treats the visual and auditory inputs as two separate channels which both perform an independent detection of the stimulus when either the visual or the auditory component crosses the threshold (λ). Both channels are contaminated with independent noise. **(B)** In the *Linear Sum* model visual and auditory signals are integrated using linear summation. Independent early noise is added to the sensors and the two signals are summed using a weighted linear sum (Σ) after which a single multisensory threshold (λ) determines whether a response is made. **(C)** The *Non-linear Sum* model adds a multiplication factor (lightning symbol) to the visual and auditory summation. Models adapted from Jones (2016). **(D)** Model fits to the average behavioral data of all mice (ALL) quantified as variance accounted for (VAF), higher values indicate a better model fit. Fits for the behavioral data of individual mice are also shown, the data of four mice show a significantly better fit for the *Linear Sum* (purple) and the *Non-linear Sum* (red) compared to the *Or* model (blue; one-way ANOVA with *post hoc* Tukey–Kramer, ^∗∗∗^*p* < 0.001).

Multisensory gain of stimulus detection is not necessarily the result of cue integration; when two stimuli are presented concurrently, the chance of detecting this compound stimulus increases also without the need of integrating these inputs (Miller, 1982). This may occur simply because stimuli from different modalities can be detected independently – yet simultaneously – by the corresponding sensory systems. We assessed whether the multisensory gain was dependent on cue integration by testing how well three models of cue-combination based on signal detection theory would fit our behavioral data (Jones, 2016; **Figures 5A–C**). The *Or* model assumes a behavioral response when either the visual component or the auditory component crosses its corresponding threshold (**Figure 5A**). Alternatively, the *Linear Sum* (**Figure 5B**) and *Non-linear Sum* (**Figure 5C**) models entail cue integration for a behavioral response to happen, which is operationalized as the summation of the visual and auditory signal distributions into a combined audiovisual decision variable with a single threshold.

The three models were fitted to the behavioral data using a cross-validation procedure and the goodness of fit of each model was determined as the variance accounted for (VAF). Fitting the model on the average performance of all mice revealed that there was a significantly better fit for the integration models compared to the *Or* model (ANOVA with *post hoc* Tukey–Kramer, *F*_2,447_ = 178.5, *p* < 10^-9^; **Figure 5D**, ALL). When fitting the models on the behavioral data for each mouse separately, four out of seven mice showed a significantly better fit for the integration models compared to the *Or* model (ANOVA with *post hoc* Tukey–Kramer; M034: *F*_2,897_ = 33.9, *p* < 10^-14^, M035: *F*_2,897_ = 32.9, *p* < 10^-14^, M038: *F*_2,897_ = 80.3, *p* < 10^-32^, M042: *F*_2,897_ = 11.0, *p* < 10^-4^; *n* = 300 cross-validated fits). Notably, there was no significant difference between the linear and non-linear sum integration models. Therefore, allowing the model to perform either sub- or supra-additivity did not increase its fit on the behavioral data indicating that these mice may well use a linear combination of visual and auditory input during multisensory stimulus detection. For three other mice, each of the models fitted the behavioral data equally well, leaving the operation most likely accountable for the behavioral benefit undetermined.

## Discussion

We developed a novel behavioral stimulus detection paradigm to probe multisensory cue-integration in head-fixed mice. Psychophysical assessment of the behavior indicated a significant detection benefit in the audiovisual compared to both unisensory conditions, which was largest when the two unisensory constituents were presented around their perceptual thresholds. Furthermore, we showed that the cross-modal enhancement of detection performance was the result of integrating visual and auditory input according to a linear sum, at least in a majority of mice. Integrating audiovisual stimuli resulted in an increase in perceptual performance that consisted of two factors; this was reflected in lower perceptual thresholds and a steepening of the psychometric function, compared to unisensory stimulation.

### Stimulus Detection as a Behavioral Readout for Multisensory Integration

Signal detection paradigms have been extensively investigated in the context of unisensory processing (Busse et al., 2011; Histed et al., 2012; Peters et al., 2014; Montijn et al., 2015; Kwon et al., 2016) but are less common in the framework of multisensory processing (but see Gingras et al., 2009; Hollensteiner et al., 2015). The first major advancement of the current set up is that mice run a large amount of trials on a daily basis for several weeks (2004 ± 142 trials in total per animal). This results in sufficient data per mouse to make psychophysical assessments for unisensory and multisensory stimulus configurations. Secondly, this setup systematically calibrates the stimulus amplitudes to the sensory capacities of the individual subject by running two adaptive staircase procedures (for visual and auditory stimuli). These procedures are robust to inter-animal variability of detection thresholds and preclude the necessity to *a priori* define stimulus intensities without knowledge of the detection thresholds of the individual animal which, especially in the auditory domain, can vary between animals (see **Figures 2F,H**). Lastly, this behavioral paradigm is compatible with neurophysiological recording methods to investigate neuronal mechanisms supporting cross-modal signal detection on the single neuron, microcircuit and systems level. Because mice are head-restrained, their location is fixed relative to the screen and speaker, the spatiotemporal arrangement of stimulus presentation with respect to the relevant sensory organs can be controlled. Furthermore, the use of psychophysics in a detection task combined with a perturbation of brain functioning can shed light on the causal role of cortical areas which process sensory information (Glickfeld et al., 2013).

In general, stimulus detection Go-No Go paradigms may suffer from impulsive behavior. This is reflected by an enhanced rate of spontaneous licking unrelated to the stimulus presentation which may result in inadvertent reward delivery. Non-stimulus related responding is assessed in this set up by inserting blank trials during which no stimulus is presented. The percentage of blank trials to which a response is registered provides the false alarm rate. Low (∼20%) spontaneous lick rates are generally not expected to influence empirical conclusions. Moreover, because spontaneous licking most likely would affect all conditions equally (**Figure 3A**), comparisons between conditions are not expected to be biased.

### Behavioral Strategies for Performing Multisensory Stimulus Detection

We showed, with fitting signal detection theoretical models to our data, that the multisensory performance enhancement is, on the group level and in a majority of individual mice, accounted for by cue integration according to a linear combination of visual and auditory inputs. This is not a trivial result because a similar performance increase can also be achieved by alternative behavioral strategies that do not involve cue integration. The redundant signal effect is an example in which an observer divides attention over two signals which both produce separate activations in the brain that are not integrated in any way (Miller, 1982). The *Or* model that we tested, which represents such a behavioral strategy, resulted in a significantly worse fit compared to cue integration models on the group level and for a majority individual mice (4 out of 7; **Figure 5**). The behavioral data of the other three mice fitted equally well to the *Or* and *(Non-) Linear Sum* models, indicating that it is impossible to distinguish between the mechanisms by which stimuli are processed. Altogether, these results render it unlikely that in our task mice generally adopt a strategy in which the sensory signals are processed separately.

We found that multisensory integration resulted in increased perceptual sensitivity (lower detection threshold) and increased acuity (steeper psychometric slope) during multi- versus unisensory detection. This is the first report, to our knowledge, to show this two-sided impact of multisensory integration on the performance of perceptual systems. Audiovisual stimulation was shown to increase sensitivity but not slope of the psychometric function in human subjects (Lippert et al., 2007), and an audiovisual detection paradigm in ferrets showed improvement of detection thresholds but did not report on changes in slope (Hollensteiner et al., 2015). The observation that both threshold and slope are impacted by audiovisual stimulation indicates that an integrative process is underlying this cross-modal benefit, instead of a general increase in sensory gain, which has been shown to solely impact threshold and not slope (Gleiss and Kayser, 2013).

### Optimal Conditions for Multisensory Integration

Multisensory integration has classically been defined according to a set of principles which govern behavioral and neuronal signals conveying multisensory stimuli. Spatial and temporal rules state that only cues that occur in close spatiotemporal proximity (i.e., from the same object) will be integrated (Slutsky and Recanzone, 2001; Stein and Stanford, 2008; Murray and Wallace, 2011). Our set up was designed in compliance with these principles; stimulus constituents were presented concurrently from a speaker and screen that were adjacent (∼20 cm distance between center of the screen and the speaker). Indeed, our behavioral data presented here indicated that mice integrate the stimuli. Furthermore, we have previously shown with using this hardware configuration that visual and auditory cues interact at the neuronal level (Meijer et al., 2017). Therefore, this setup offers the ideal setting to study a third principle; that the multisensory enhancement is inversely related to the effectiveness by which the unisensory cues drive the sensory systems. This principle was originally conceived as a framework to describe responses from single neurons recorded from the superior colliculus of the cat (Meredith and Stein, 1983, 1986) but has since been applied to multisensory cue integration on the behavioral level and on the neuronal level on the cortex (Serino et al., 2007; Gleiss and Kayser, 2012). However, because potential pitfalls in the quantification of this effect have been pointed out, existing evidence in support of this principle is potentially less substantial than assumed (Holmes, 2007, 2009). We found that the maximal multisensory behavioral increase is achieved when both stimulus constituents are of equal subjective intensity and presented at their perceptual thresholds (**Figures 3C,D**). Consistent with the principle of inverse effectiveness, the magnitude of the gain decreased as the stimulus components become more salient. The magnitude of the gain, however, strongly diminished when amplitudes of the stimulus constituents were below threshold. Thus, even for paired stimulus constituents which elicited response rates of ∼40% in unisensory conditions, the multisensory gain was almost negligible. Single neurons and neural networks transform synaptic input into spiking output in a non-linear fashion (Roxin et al., 2011), and this non-linearity can be further increased at the population level by resonant enhancement of neural activity (Knight, 1972). Our behavioral data suggest that a unisensory stimulus presented near the perceptual threshold may induce a synaptic input to the local network resulting in a maximal non-linear input-output transformation. The presentation of two concurrent near-threshold stimuli of different modalities, each impacting on the steepest part of their respective input-output curves, may then lead to a further amplification of population spiking activity. This hypothesis can be tested using intracellular measurements in, for example the primary sensory cortex, or computational modeling. Thus, the multisensory behavioral enhancement we report here is consistent with the principle of inverse effectiveness when stimulus intensities are in the range between their perceptual thresholds and full contrast and quickly drops when stimulus intensities are below the perceptual thresholds. In its entirety, the relation between stimulus intensity and multisensory gain is in line with data obtained with multisensory two-alternative forced choice paradigms, which indicate that the largest behavioral gain is when unisensory stimuli are at the point of subjective equality (Ernst and Banks, 2002; Gu et al., 2008; Raposo et al., 2012).

To conclude, in this study we developed and tested a behavioral paradigm for head-fixed mice to investigate multisensory cue integration based on signal detection. This paradigm is an important addition to the existing suite of multisensory signal detection tasks because it allows for stimulus adjustments to the abilities of the individual subject and allows for sufficient trials run to perform psychophysical assessments. It is also an important addition to the available repertoire of multisensory paradigms for rodents, which mostly consists of stimulus discrimination tasks (Raposo et al., 2012; Nikbakht et al., 2018). The increase in development of behavioral tasks in recent years has accelerated our understanding of multisensory processing because it created the circumstances needed to assess the top-down influences of behavioral constraints and cognitive processes on multisensory processing, which is not possible in anesthetized or passively observing awake animals in which multisensory processing is traditionally studied. During multisensory stimulus detection and discrimination observers use cue-integration, although the nature of the underlying process of integration is most likely different. What the different ways of employing multisensory cue-integration entail on the neuronal level is subject to further neurophysiological research.

## Data Availability

The datasets generated for this study are not publicly available because they are subject to further analysis. Requests to access the datasets should be directed to CL, c.s.lansink@uva.nl.

## Author Contributions

GM, CP, and CL designed the research, analyzed and interpreted the data, and wrote the manuscript. GM and TD performed the experimental work. JP contributed to analysis tools.

## Conflict of Interest Statement

The authors declare that the research was conducted in the absence of any commercial or financial relationships that could be construed as a potential conflict of interest.
